# A dynamic model for delta rhythm fit to high-frequency cortical activity data shows discrete functional connectivity in mouse cortex

**DOI:** 10.1186/1471-2202-15-S1-P82

**Published:** 2014-07-21

**Authors:** Mark A Reimers, Majid Mohajerani, Timothy H Murphy

**Affiliations:** 1 Department of Psychiatry, Virginia Commonwealth University, Richmond, VA 23221, USA; 2 Canadian Center for Behavioral Neuroscience, University of Lethbridge, Lethbridge, AB, Canada; 3 Department of Psychiatry, University of BC, Vancouver, BC, Canada

## 

Spontaneous activity as recorded by fMRI has often been used to infer active connections ('functional connectivity') in the human brain through correlations of activity measures. Some serious questions have been raised about the interpretation of these correlations, which are often apparent only on time scales of tens of seconds. Confirmation of correlations in measures of activity on shorter time-scales closer to those of neural activity would be very desirable.

Numerous mechanisms have been proposed for various rhythms but in the past half-century little consensus has been reached on the mechanism of any major rhythm. The recent development of high-throughput imaging methods enable us for the first time to rigorously and quantitatively test ideas about the dynamics of brain rhythms.

**Figure 1:**
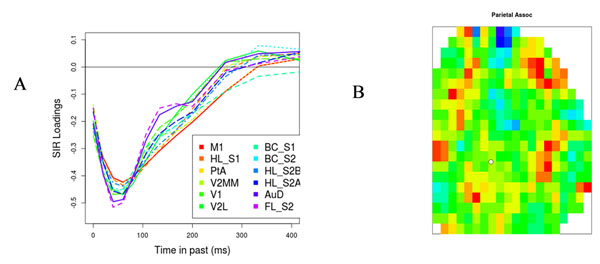
A (left) the dependence of change in voltage on previous voltage is identical for all cortical regions; time in past is represented on the x-axis. B (right). The estimated functional connectivity from the right top surface of mouse cortex into the parietal association area: red represents high connectivity; green zero; blue negative.

We have generated high-resolution data on neural activity over most of one hemisphere of mouse cortex by voltage-sensitive dyes, in both anesthetized and awake animals. In previous work [1] we have analyzed relations between activity measures at different locations in terms of correlations. Here we fit these data to a predictive model, in which we attempt to predict the next change in activity at every point on cortex from the current pattern of activity over cortex. We fit both linear and non-linear models, whose parameters represent the intrinsic dynamics of local cortical regions and the inputs from distal regions. We find that all regions of mouse cortex appear to have virtually identical patterns of intrinsic dynamics (Figure 1A). We find that even a simple linear fit gives surprisingly sparse patterns of inferred connectivity. Where we have clear anatomical information, these fitted patterns appear to match known anatomy. Furthermore this fit can be used to identify the most prominent functional inputs into anatomically diffusely-connected areas such as the parietal association area (Figure 1B).

Our results suggest that similar methods can be used with data generated by the new high-throughput technologies anticipated by the BRAIN initiative, to understand communication between brain regions at time scales of neural computation.
